# DNA Methylation Variation Is a Possible Mechanism in the Response of *Haemaphysalis longicornis* to Low-Temperature Stress

**DOI:** 10.3390/ijms232315207

**Published:** 2022-12-02

**Authors:** Chuks Fidelis Nwanade, Zihao Wang, Ruwei Bai, Ruotong Wang, Tianai Zhang, Jingze Liu, Zhijun Yu

**Affiliations:** Hebei Key Laboratory of Animal Physiology, Biochemistry and Molecular Biology, Hebei Collaborative Innovation Center for Eco-Environment, Hebei Basic Subject Research Center for Cell Biology, Ministry of Education Key Laboratory of Molecular and Cellular Biology, College of Life Sciences, Hebei Normal University, Shijiazhuang 050024, China

**Keywords:** *Haemaphysalis longicornis*, epigenetic regulation, DNA methylation, whole-genome bisulfite sequencing, low-temperature stress

## Abstract

Ticks are notorious ectoparasites and transmit the greatest variety of pathogens than any other arthropods. Cold tolerance is a key determinant of tick abundance and distribution. While studies have shown that DNA methylation is one of the important epigenetic regulations found across many species and plays a significant role in their response to low-temperature stress, its role in the response of ticks to low-temperature stress remains unexplored. Herein, we explored the DNA methylation profile of the tick, *Haemaphysalis longicornis,* exposed to low-temperature stress (4 °C) using whole-genome bisulfite sequencing (WGBS). We found that approximately 0.95% and 0.94% of the genomic C sites were methylated in the control and low-temperature groups, respectively. Moreover, the methylation level under the CG context was about 3.86% and 3.85% in the control and low-temperature groups, respectively. In addition, a total of 6087 differentially methylated regions (DMRs) were identified between the low-temperature and control groups, including 3288 hypermethylated and 2799 hypomethylated DMRs. Further, Gene ontology (GO) and Kyoto Encyclopedia of Genes and Genomes (KEGG) pathway analysis of differentially methylated genes revealed that most of the DMGs were significantly enriched in binding and RNA transport pathways. Taken together, this research confirmed, for the first time, the whole genome DNA methylation profile of *H*. *longicornis* and provided new insights into the DNA methylation changes relating to low-temperature stress in *H*. *longicornis*, as well as provided a foundation for future studies on the epigenetic mechanism underlying the responses of ticks to abiotic stress.

## 1. Introduction

*Haemaphysalis longicornis*, also known as the longhorned tick, is one of the most prevalent tick species in eastern Asia and an exotic species in Australia, New Zealand, and the United States [1,2]. It is a three-host tick that feeds on multiple hosts, including domestic and wild animals [3], and occasionally attacks humans [4,5]. Its veterinary and medical importance has been widely recognized because of its vector ability in transmitting a great diversity of pathogens, including severe fever with thrombocytopenia syndrome virus, *Anaplasma phagocytophilum*, *Babesia microti*, *Ehrlichia chaffeensis*, *Rickettsia raoultii* and *Theileria orientalis* [1], which can cause severe diseases in animals and humans [6]. The *H. longicornis* ticks spend most of their life cycle off the host, where they are exposed to many factors, including abiotic factors [7]. Low temperature is an important abiotic factor that can affect the survival and development of *H*. *longicornis* off the host [8]. However, the unfed adult and nymphal ticks of *H*. *longicornis* are capable of overwintering in the field [7,8,9], and they can employ various techniques to ensure their survival under low-temperature stress, which is a process called cold hardiness [9].

The response of plants and animals to abiotic stress is often accompanied by epigenetic modifications [10]. These are changes in gene expression that are not related to changes in DNA sequence. In eukaryotes, 5-methylcytosine (5 mC) is one of the best-studied DNA methylation (epigenetic mechanism) [11]. However, several forms of DNA methylation, including N6-methyladenine (6 mA) modification have been observed [11]. Increasing evidence has shown that DNA methylation is involved in the regulation of gene expression in response to abiotic stress in many species including *Nile tilapia* [12], Zebrafish [13], *Hevea brasiliensis* [14], *Bombyx mori* [15], and *Capsicum annuum* [16]. While a number of DNA methyltransferase (Dnmts) genes have been implicated in the cold tolerance of *H*. *longicornis* and *Dermacentor silvarum* [17], genome studies are necessary to expand our knowledge and to understand the role of DNA methylation in the response of ticks, including *H*. *longicornis* to abiotic stress. Moreover, genomic methylation has not been well studied in ticks.

Herein, we investigated the DNA methylation profile of *H*. *longicornis* exposed to low-temperature stress using whole genome bisulfite sequencing, hoping to expand our knowledge of the physiological regulations related to cold tolerance in *H*. *longicornis*.

## 2. Results

### 2.1. Summary of Bisulfite Sequencing and DNA Methylation

As indicated in our previous study, six days is sufficient for *H*. *longicornis* to respond to low temperature (4 °C) [17]. In the present study, to explore the role of DNA methylation in *H*. *longicornis* response to low temperature, we sequenced three replicates of *H*. *longicornis* adults exposed to 4 °C for six days using whole-genome bisulfite sequencing (WGBS). As shown in Table 1, mean raw reads of 421,797,949 (126.54 Gb) and 427,937,671 (128.38 Gb) were obtained by WGBS from *H*. *longicornis* adults exposed to low temperature (4 °C) and the control group (adults maintained at a rearing temperature of 26 °C), respectively. After quality control and filtering, about 410,432,711 (111.33 Gb) and 418,108,642 (114.01 Gb) clean reads were obtained from the two groups of adults, respectively, of which 38.70% (control) and 38.56% (treatment) clean reads were uniquely mapped to the *H*. *longicornis* tick reference genome [18] with a duplication rate of 11.94% and 11.72%, respectively. Moreover, the bisulfite conversion rates exceeded 99.43% in both groups, indicating the credibility and accuracy of WGBS.

To further explore the DNA methylation levels, methylated cytosine levels of the three sequence contexts: CG, CHG, and CHH (where H = A, T, or C) were calculated. We observed that about 0.95% of the genomic C sites were methylated in the control group, whereas about 0.94% of the genomic C sites were found in the group exposed to a low temperature. Further, the methylation level under the CG context was about 3.86% in the control group and 3.85% in the group exposed to a low temperature. We also observed 0.07% and 0.08% methylation levels in the CHG context in the control group and low-temperature exposed group, respectively. However, the methylation level under the CHH context was 0.03% in both groups (Table S1).

### 2.2. Sample Correlation and Cluster Analysis

**Figure 1:**
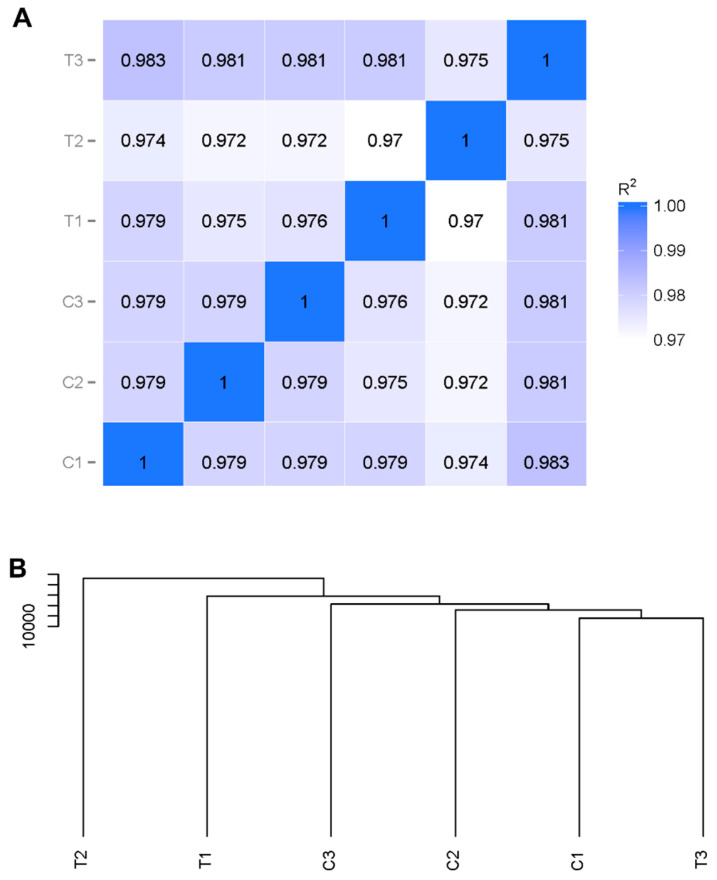
The clustering of the *H. longicornis* adults with or without treatment at 4 °C for six days. (**A**). Pearson correlation based on CG context among the replicate samples. (**B**). Sample CG context clustering dendrogram. R^2^: Pearson’s correlation coefficient, T: low-temperature treatment (4 °C for 6 days); C: control group (26 °C for 6 days).

The correlation of the level of methylation among samples is an essential indicator to assess the reliability of the study. Herein, the Pearson correlation based on the CG context was conducted among the replicate samples of control and low temperature. As shown in Figure 1A, Pearson’s correlation was constantly 0.979 within control samples and in the range of 0.972–0.981 within low-temperature samples. However, the coefficient ranged from 0.972–0.983 between both samples. Further, the cluster analysis partially separated the control group replicates from the low temperature group replicates (Figure 1B).

### 2.3. DNA Methylation Levels of the Functional Regions

**Figure 2:**
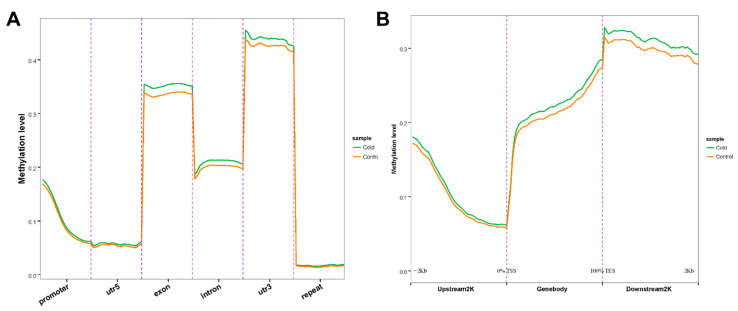
DNA methylation profiles of *H*. *longicornis* adults at different genomic functional rgions. (**A**). Distribution of the methylation level of functional regions between the control and low-temperature mCG/CG. (**B**). Distribution of methylation level of genes upstream and downstream between the control and low-temperature mCG/CG. TSS: transcription start site, TES: transcription end site.

The distribution of DNA methylation was evaluated in various genomic functional regions (promoters, 5′ untranslated region (UTR), exons, introns, 3′ UTR, and repeat regions) on CG sites (Figure 2). We found that the methylation level was the highest in 3′ UTR regions, followed by exon, intron, and promoter regions, and relatively low in 5′ UTR and repeat regions. Interestingly, the methylation levels were higher in the low-temperature group than in the control group in all functional regions, especially in the 3′ UTR and exon regions. Moreover, methylation peaked in the downstream region near the transcription end site (TES).

### 2.4. Differentially Methylated Regions Analysis

**Figure 3:**
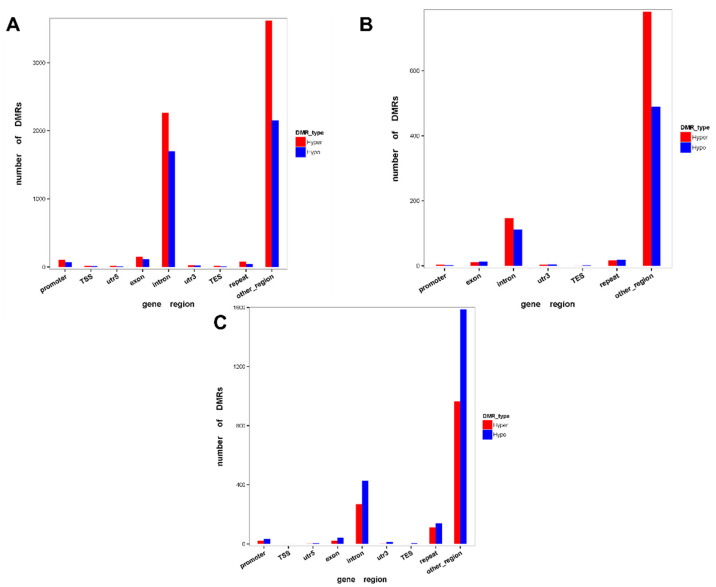
DMRs methylation levels of the *H*. *longicornis* adults with or without treatment at 4 °C for six days. (**A**). Distribution of cold vs. control CG DMRs in different regions of the genome. (**B**). Distribution of cold vs. control CHG DMRs in different regions of the genome. (**C**). Distribution of cold vs. control CHH DMRs in different regions of the genome. TES: transcription end site, TSS: transcription start site.

**Figure 4:**
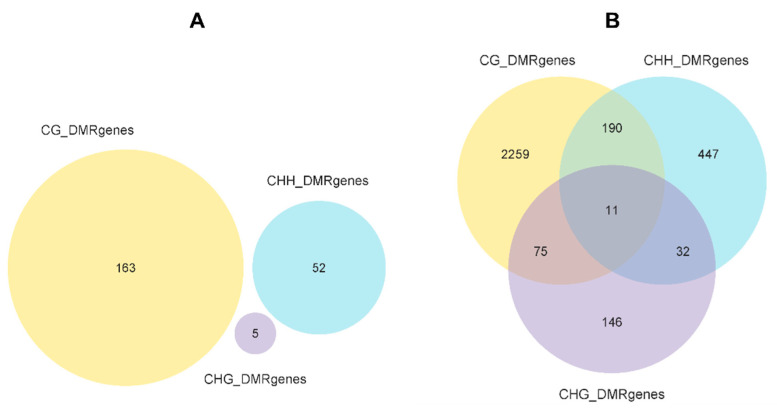
Venn analysis of cold vs. control DMGs under CG, CHG, and CHH contexts in the promoter and gene body region of the *H. longicornis* adults. (**A**). Venn analysis of DMGs in the promoter region. (**B**). Venn diagrams of DMGs in the gene body region.

**Figure 5:**
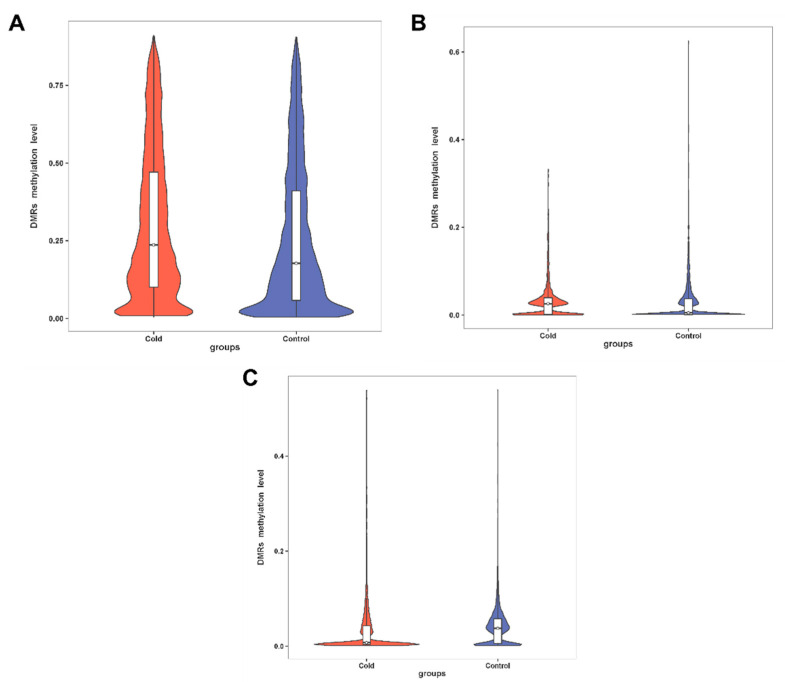
Boxplot analysis of the methylation level of DMRs in the cold vs. control group of the *H*. *longicornis* adults. (**A**). Boxplot analysis of the CG DMR methylation level. (**B**). Boxplot analysis of the CHG DMR methylation level. (**C**). Boxplot analysis of the CHH DMR methylation level.

To further determine the role of methylation in response to low-temperature stress, we examined DMRs and differentially methylated genes (DMGs) of cold-stressed *H*. *longicornis* adults compared to those exposed to rearing temperature. A total of 6087 DMRs were identified in cold-stressed vs. rearing temperature groups (Table S2), including 3288 hypermethylated and 2799 hypomethylated DMRs (Tables S3 and S4). Moreover, hypermethylated DMRs located in promoters, TSS, 5′ UTR, exon, intron, 3′ UTR, TES, repeat, and other regions were more than that of hypomethylated DMRs in the CG context (Figure 3A), but less than that of hypomethylated DMRs in exon, 3′ UTR, TES, and repeat in the CHG context (Figure 3B). While CHH hypomethylated DMRs were more than that of hypermethylated DMRs in the promoter, exon, intron, 3′ UTR, TES, repeat, and other regions (Figure 3C). The DMGs under CG, CHG, and CHH contexts in the promoter and gene body region are presented in Figure 4. The results showed that a total of 163, 5 and 52 DMGs under CG, CHG and CHH contexts, respectively, were observed in the promoter region (Figure 4A). The results also showed that 2259, 146 and 447 DMGs under CG, CHG, and CHH contexts, respectively, were observed in the gene body region, while 11 DMGs were overlapping among the three contexts (CG, CHG, and CHH) (Figure 4B). Further, the results of the boxplot analysis of DMRs showed that the methylation level of the cold-stress group was higher than that of the control group in the CG and CHG contexts (Figure 5). Further observations indicated that the methylation level of CG was the highest (Figure 5A).

### 2.5. Gene Ontology (GO) and Kyoto Encyclopedia of Genes and Genomes (KEGG) Pathway Enrichment Analysis

**Figure 6:**
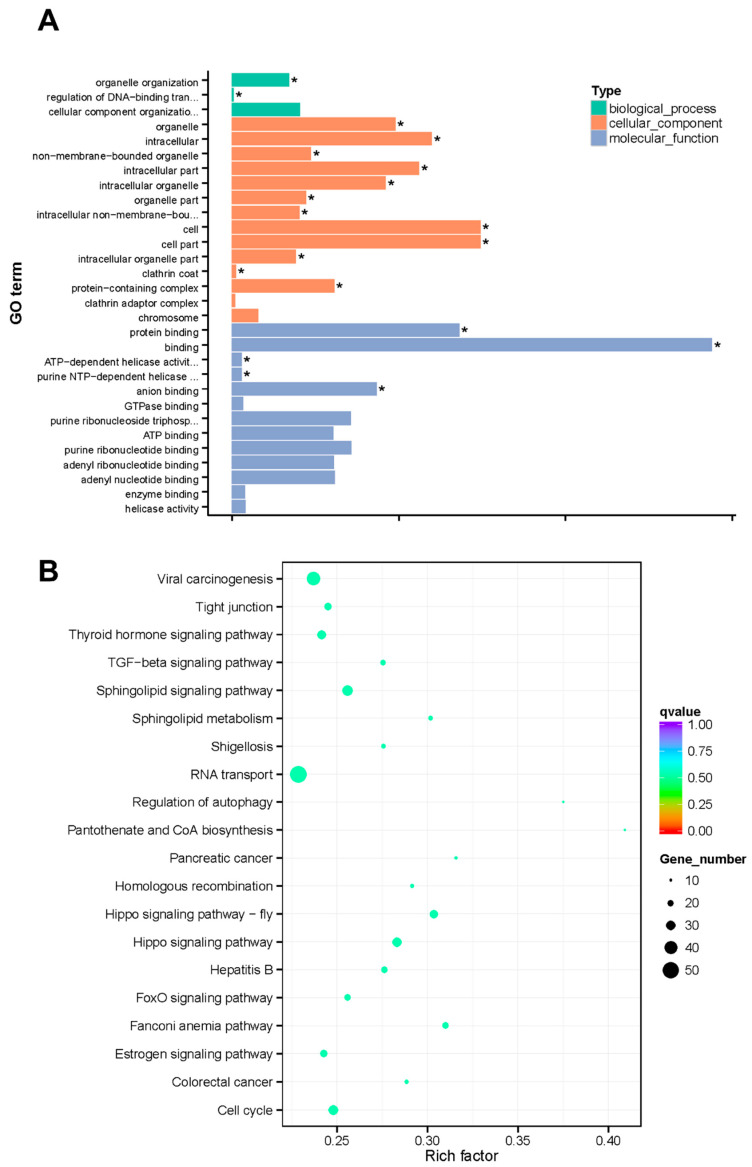
GO and KEGG enrichment analyses of cold vs. control CG DMR genes of the *H*. *longicornis* adults. (**A**). Most enriched GO terms. (**B**). KEGG pathway enrichment. * Significantly enriched (corrected *p* < 0.05).

GO and KEGG analyses were performed to explore DNA methylation changes under low-temperature stress. Since most of the DMGs were in the CG methylation context, DMG functional enrichment analyses were performed based on CG methylation. The DMGs were assigned to three categories: biological process, cellular component, and molecular function. Our results revealed that 19 GO terms were significantly enriched (corrected *p* < 0.05) in the control vs. low-temperature groups, and 12 of them were classified as having a cellular function (Figure 6A). Specifically, these DMGs were involved in the following GO terms: Binding, cell, cell part, protein binding, intracellular, intracellular part, organelle, intracellular organelle, anion binding, protein-containing complex, and other processes. To provide more insight into the pathways, a KEGG pathway analysis of the DMGs was conducted. The results showed that the top two enriched KEGG pathways were RNA transport and viral carcinogenesis (Figure 6B).

## 3. Discussion

Ticks, including *H*. *longicornis*, are threatened with death by abiotic stress, including low-temperature stress [9]. Hence, *H*. *longicornis* can employ various techniques in response to this environmental stress. This is a complex process involving many changes, including biochemical and metabolic changes [9,18]. The molecular responses of *H*. *longicornis* to low-temperature stress, including changes in many Dnmt genes have been studied [17,19]. To expand this previous research, for the first time, we used WGBS to explore DNA methylation changes in ticks, including *H*. *longicornis*. This allows us to comprehensively study genome-wide DNA methylation profiles of this medical and veterinary important tick.

Our results showed that the methylated genomic C site in the treated group was slightly lower than that in the control group. While the difference (in the methylated genomic C between the treated and control groups) is small, it is in agreement with some previous DNA methylation studies. For example, Chen et al. found that the type 2 diabetes mellitus methylated cytosine rate was slightly lower than that in the control group [20]. A recent study showed a reduction of DNA methylation levels in sugar beet in response to a cold condition [21]. However, there are some studies with contrary results. For instance, under heat-humidity stress in silkworms, Chen et al. found that the methylated genomic C sites of the treated group were slightly higher than that of the control group [15]. Pozo et al. also found higher rates of cytosines that were methylated in a treated bumblebee [22]. These differences may be attributed, in part, to variations in treatments and/or environmental stressors. Moreover, the ability of ticks to withstand low-temperature stress is important for their survival and development, and variation in their biochemical and physiological response to cold stress has been previously demonstrated [9,17,23], which was attributed to temperature variations, different exposure times, and developmental stages.

In insects, cytosine methylation level ranges from 0–3%, about 5% in birds and mammals, 10% in amphibians and fish, and over 30% in some plants [24,25]. In agreement with previous data (following high-temperature/humidity stress in *B*. *mori*) [15], the percentage methylation (methylated genomic C site) in the present study was less than 1%. However, they are greater than the percentage methylation (0.14% and 0.12%) reported in *B*. *mori* [15], suggesting that different species and/or environmental conditions may lead to different levels of DNA methylation. Despite the fact that the DNA methylation levels in insects are much lower than that in other species, it is well known that it is a mechanism by which they respond to abiotic stress [15]. This suggests that low levels of DNA methylation may also have an important effect on ticks, including *H*. *longicornis* in response to abiotic stress.

DNA methylation can be classified into three types based on the sequence of cytosines, specifically CG, CHG, and CHH (where H = A, C, or T) [26]. Our data showed that cytosine methylation was detected in all three cytosine contexts, with the highest levels of methylation at the CG site. DNA methylation at the CG site has been discussed previously in several studies with other arthropods [15], and in general, DNA methylation usually occurs at CG sites [27]. Our cluster analysis revealed that the treatment group was partially separated from the control group, which possibly indicated that the DNA methylation of *H*. *longicornis* may not respond strongly to low-temperature stress. While further experimental verification is needed, this may be due to the temperature and exposure period used in this study. Moreover, some researchers have reported changes in DNA methylation with the extension of treatment time [17,28].

DNA methylation has been associated with many functions and methylation can take place in several locations [24]. By analyzing DNA methylation levels in different functional genomic regions at the CG site, we discovered that, in the studied tick, the methylation level was highest at the 3′ UTR, in contrast to the exon region in other invertebrates [15]. However, a recent study has shown that 3′ UTR is a functional and relevant DNA methylation site [29]. Together, the results suggested that in *H*. *longicornis* there are different functional genomic regions, and DNA methylation is more likely to occur in the 3′ UTR region, followed by the exon region in response to low-temperature stress.

We further observed a different distribution of hypermethylated and hypomethylated DMRs across the different functional genomic regions, indicating differential DNA methylation in the genomic region [30]. We found several genes differentially methylated (mostly at the CG site) between the control and low-temperature groups. While further research is needed to elucidate their functional roles, these genes may play vital roles in the processes that may be associated with low-temperature stress in *H*. *longicornis*.

Based on the GO and KEGG pathway analyses of DMGs, we found that most of the DMGs were most significantly enriched in binding and RNA transport. A previous study on DNA methylation analysis in *B*. *mori* response to abiotic stress also revealed that DMGs were most significantly enriched in binding and RNA transport [15], suggesting their potential roles in the regulation of relevant genes. DNA methylation can influence gene expression, especially in the response of organisms to environmental conditions. A previous study has indicated several possible Dnmt genes contributing to the response of the ticks, *H*. *longicornis* and *D. silvarum* to cold conditions [17]. The expression levels of the Dnmt genes such as *HIDnmt1* and *DsDnmt1* were significantly down-regulated in response to cold stress, but they were significantly up-regulated with the extension of days of low-temperature treatment [17], indicating that their expression might be beneficial to protect ticks from cold stress. Elsewhere, Chen et al. reported changes in silkworm gene expression profiles (regulated by DNA methylation) in response to high-temperature/humidity stress [15]. It has also been demonstrated that changes in DNA methylation are essential for insects to regulate gene expression [31]. It is therefore plausible that low-temperature stress can affect the expression profiles of genes related to low-temperature tolerance and induce methylation variation in *H*. *longicornis* DNA. However, the expression pattern of genes related to low-temperature tolerance in *H*. *longicornis* requires further investigation.

## 4. Materials and Methods

### 4.1. Tick Collection and Treatment

The second generation of unfed adult females of *H*. *longicornis* used in this study were established from an initial population of *H*. *longicornis* collected by flag dragging from vegetation at Xiaowutai National Nature Reserve Area (40°03′03″ N, 115°23′15″ E), Hebei Province, China. The ticks collected were fed on domestic rabbits [32] and maintained in an incubator (26 ± 1 °C, 85 ± 5% relative humidity (RH), 16:8 h (light: dark; L:D) photoperiod) during the non-feeding period. For low-temperature treatment, a group of 20 unfed females was kept in an incubator (4 °C) for 6 days. After treatment, they were transferred to a normal incubator (26 ± 1 °C, 85 ± 5% RH, 16:8 h (L:D)) and held for 24 h. Ticks kept in the normal incubator (rearing temperature) served as the control, and each treatment was replicated three times.

### 4.2. DNA Extraction

Genomic DNA was extracted using Magnetic Universal Genomic DNA Kit (Tiangen, Beijing, China) following the manufacturer’s recommendations. To meet the library preparation requirements, DNA degradation and contamination were examined by agarose gel electrophoresis (1%). The purity and concentration of DNA were verified using the NanoPhotometer^®^ spectrophotometer (IMPLEN, Calabasas, CA, USA) and Qubit^®^ DNA Assay Kit in Qubit^®^ 2.0 Fluorometer (Life Technologies, Carlsbad, CA, USA), respectively.

### 4.3. Library Preparation and Quantification

A total of 100 ng genomic DNA spiked with 0.5 ng lambda DNA was fragmented by sonication to 200–300 bp with Covaris S220 instrument (Covaris, Inc., Woburn, MA, USA). These DNA fragments were treated with bisulfite using EZ DNA Methylation-GoldTM Kit (Zymo Research), and the library was constructed by the Novogene Corporation (Beijing, China). Subsequently, pair-end sequencing of the sample was performed on the Illumina platform (Illumina, San Diego, CA, USA). Library quality was assessed on the Agilent Bioanalyzer 2100 system.

### 4.4. Data Analysis

The Illumina Novaseq platform was used to sequence the library, which generated 125 bp/150 bp paired-end reads. An Illumina CASAVA pipeline was used for image analysis and base calling, and the final 125 bp/150 bp paired-end reads were generated. The sequenced raw data have been deposited in the National Center for Biotechnology Information (NCBI) Sequence Read Archive (SRA) with the accession number PRJNA893382.

### 4.5. Quality Control

FastQC (fastqc_v0.11.5) was used to perform basic statistics on the raw read quality. The reads sequences in FASTQ format generated by the Illumina pipeline were then pre-processed with the parameters (SLIDINGWINDOW: 4:15; LEADING:3, TRAILING:3; ILLUMINACLIP: adapter.fa: 2: 30: 10; MINLEN:36) via Trimmomatic (Trimmomatic-0.36) software. The remaining reads that passed all the stages of filtration were considered to be clean reads and all subsequent analyses were based on these clean reads. Finally, we used FastQC to conduct basic statistics on the quality of the clean data (clean reads).

### 4.6. Reference Data Preparation before Analysis

Prior to the analysis, reference data for the tick species, including the reference sequence fasta file, the annotation file in gtf format, the GO annotation file, the description file, and the gene region file in bed format were prepared. Regarding the bed files, we predicted repeats through RepeatMasker (http://www.repeatmasker.org/cgi-bin/WEBRepeatMasker) and then obtained the CGI track from a genome using cpgIslandExt of the UCSC table browser.

### 4.7. Reads Mapping to the Reference Genome

Bisulfite-treated reads alignments with the reference genome (−X 700—tail column) were conducted with Bismark software (version 0.16.3) [33]. The reference genome was initially converted to a bisulfite-converted version (C-to-T and G-to-A converted) and subsequently indexed with bowtie2 [34]. Sequence reads were also completely converted to bisulfite-converted versions (C-to-T and G-to-A converted) before aligning them with similar transformed versions of the genome in a directional fashion. The normal genomic sequence was then used for comparison with sequence reads that generated the greatest and unique alignment from the two alignment processes (original above and lower strands), and the state of the methylation of all cytosine positions in the read was deduced. Reads that aligned to the same genomic locations were considered to be duplicated reads. The sequencing coverage and depth were summarized with deduplicated reads.

Methylation extractor (bismark_methylation_extractor-no_overlap) results were converted to bigWig format for visualization with the IGV browser. The non-conversion rate of sodium bisulfite was computed as the cytosine percentage sequenced in the reference positions of cytosine in the lambda genome.

### 4.8. Estimation of Methylation Level

To determine the site of methylation, the total methylated counts (mC) were modeled as a binomial (Bin) random variable with methylation rate r

mC~Bin (mC + umC r)

The level of methylation of the sequence was calculated by diving the sequence into multiple bins with 10 kb size. The total methylated and unmethylated read counts were calculated in each window. The level of Methylation (ML) for each window or C position indicates the methylated Cs fraction and is stated as:

$$\mathrm{ML}\left(C\right)=\frac{\mathrm{reads}\left(\mathrm{mC}\right)}{\mathrm{reads}\left(\mathrm{mC}\right)+\;\mathrm{reads}\left(C\right)}$$

The calculated ML was then corrected using a bisulfite non-conversion rate, as stated in previous research [35]. Taking into account the non-conversion rate r of bisulfite, the corrected ML was evaluated as follows:

$$\mathrm{ML}\left(\mathrm{corrected}\right)=\frac{\mathrm{ML}-\;r}{1-\;r}$$

### 4.9. Differentially Methylated Analysis

Differentially methylated regions (DMRs) were determined with DSS software [36,37,38]. DMRs were identified by the following parameters: Smoothing = TRUE, smoothing. span = 200, delta = 0, p.threshold = 0.00001, minlen = 50, minCG = 3, dis.merge = 100, pct.sig = 0.5. According to the distribution of DMRs in the genome, genes associated with DMRs were defined as genes whose regions of the gene body (from TSS to TES) or the promoter region (upstream of TSS) overlaps with the DMRs.

### 4.10. GO and KEGG Enrichment Analysis of DMR-Related Genes

Gene Ontology (GO) enrichment analysis of DMR-associated genes was carried out by GOseq R package [39], in which gene length bias was corrected. GO terms with corrected *p*-value less than 0.05 were considered significantly enriched with genes related to DMR. Kyoto Encyclopedia of Genes and Genomes (KEGG) [40] is a database resource for understanding the advanced functions and utilities of biological systems, including large-scale molecular datasets produced by genome sequencing [41]. To test the statistical enrichment of genes related to DMR in the KEGG pathways, KOBAS software [42] was utilized.

## 5. Conclusions

In this study, we explored the methylation profile of H. longicornis exposed to low-temperature stress and normal conditions by WGBS. Our results revealed changes in DNA methylation patterns, DMRs, DMR-related genes, processes, and pathways that may be involved in H. longicornis response to environmental stressors. While this study has some limitations, for instance, the expression profile of the key genes has not been validated and the interpretation of our results may be restricted by exposure temperature and/or time, as well as sample size. Nevertheless, our research is significant in that it enhanced our knowledge and provided new insights into DNA methylation changes relating to low-temperature stress in H. longicornis, as well as provided a foundation for future studies of the epigenetic mechanism underlying the responses of ticks to abiotic stress.

## Figures and Tables

**Table 1:** Summary of bisulfite sequencing.

	Samples	
	Control	Treatment
Raw reads	427,937,671	421,797,949
Raw bases (G)	128.38	126.54
Clean reads	418,108,642	410,432,711
Clean bases (G)	114.01	111.33
Clean ratio (%)	88.81	87.97
Q20 (%)	96.63	96.75
Q30 (%)	90.15	90.39
GC content (%)	24.48	24.59
Bisulfite conversion rate	99.466	99.433
Mapped reads	161,808,422	158,247,367
Unique mapping rate (%)	38.70	38.56
Duplicate rate (%)	11.94	11.72

## Data Availability

The sequenced raw data have been deposited in the National Center for Biotechnology Information (NCBI) Sequence Read Archive (SRA) with the accession number PRJNA893382.

## Supplementary Materials

The following supporting information can be downloaded at: https://www.mdpi.com/article/10.3390/ijms232315207/s1.
